# Injectable Autocatalytic Hydrogel Triggers Pyroptosis to Stimulate Anticancer Immune Response for Preventing Postoperative Tumor Recurrence

**DOI:** 10.1002/advs.202408415

**Published:** 2024-10-28

**Authors:** Zhiping Rao, Yutong Zhu, Zhuang Chen, Yi Luo, Zuo Yang, Weijing Liu, Chaoqiang Qiao, Yuqiong Xia, Peng Yang, Dong‐Man Ye, Zhongliang Wang

**Affiliations:** ^1^ Lab of Molecular Imaging and Translational Medicine (MITM) Engineering Research Center of Molecular & Neuroimaging Ministry of Education School of Life Science and Technology Xidian University & International Joint Research Center for Advanced Medical Imaging and Intelligent Diagnosis and Treatment Xi'an Shaanxi 710126 P. R. China; ^2^ Medical college Xi'an International University Xi'an Shaanxi 710077 P. R. China; ^3^ Department of Medical Imaging Cancer Hospital of China Medical University Liaoning Cancer Hospital & Institute Shenyang Liaoning 110042 P. R. China

**Keywords:** hydrogel, immune response, postsurgical recurrence, pyroptosis, triple‐negative breast cancer

## Abstract

Modulating immunosuppression while eliminating residual microscopic tumors is critical for inhibiting the postoperative recurrence of triple‐negative breast cancer (TNBC). Although immunotherapy has shown potential in achieving this goal, due to multiple immunosuppression and poor immunogenicity of apoptosis, a satisfactory anti‐recurrence effect still faces the challenge. Herein, an injectable hydrogel‐encapsulated autocatalytic copper peroxide (CP@Gel) therapeutic platform is designed and combine it with the clinical‐grade DNA methyltransferase inhibitor decitabine (DAC) to effectively inhibit TNBC growth and postoperative recurrence via pyroptosis, killing residual cancer cells that bypass apoptosis resistance while also improving immunogenicity and modulating immunosuppression to achieve an intense anti‐tumor immune response. Following injection of the CP@Gel, the sustained release of CP leads to the autocatalytic generation of reactive oxygen species, resulting in caspase‐3 activation, and the pre‐administered DAC inhibits the methylation of *Gsdme* to elevate the GSDME protein levels, leading to intense pyroptosis and anti‐tumor immune responses. The in vivo results show a 67% elimination of local tumor recurrence via treatment with DAC+CP@Gel, suggesting the successful integration of sustained drug release with autocatalysis and epigenetic modification. The results thus suggest great potential for pyroptosis‐based and injectable hydrogel‐aided strategies for preventing the postoperative recurrence of TNBC.

## Graphical abstract

This work develops an injectable hydrogel‐encapsulated autocatalytic copper peroxide (CP@Gel) therapeutic platform and combines it with the clinical‐grade DNA methyltransferase inhibitor decitabine (DAC) to effectively inhibit TNBC growth and postoperative recurrence via evoking pyroptosis and immune response, which suggesting great potential for pyroptosis‐based and injectable hydrogel‐aided strategies for preventing the postoperative recurrence of TNBC.

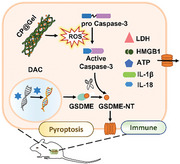

## 1 Introduction

Surgical resection is the primary treatment for triple‐negative breast cancer (TNBC).^[^ ^1^ ^]^ However, the long‐term prognosis of patients with TNBC following surgery remains unsatisfactory due to a high rate of postsurgical recurrence.^[^ ^2^ ^]^ Studies have demonstrated that the main cause of postoperative recurrence is residual microscopic lesions that cannot be eliminated during surgery.^[^ ^3^ ^]^ Additionally, increasing evidence suggests that surgery is likely to induce a short period of immunosuppression, allowing the expansion and escape of residual tumor cells while also inhibiting the activity of antitumor leukocytes,^[^ ^4^ ^]^ including dendritic cells (DCs) and cytotoxic T lymphocytes.^[^ ^2b^ ^]^ Therefore, to effectively inhibit the postoperative recurrence of TNBC, strategies that can simultaneously modulate immunosuppression while eliminating residual microscopic disease is urgently required.

Recently, advances in immunotherapy have shown an array of light to achieve the goal of preventing postoperative recurrence.^[^ ^5^ ^]^ However, the multiple immunosuppression and poor immunogenicity associated with tumor apoptosis^[^ ^6^ ^]^ renders satisfactory anti‐tumor immune activation a considerable challenge. Therefore, the introduction of a new pattern of cell death with high immunogenicity instead of apoptosis is highly desirable. Pyroptosis is a newly defined proinflammatory and immunogenic cell death that is characterized by membrane perforation, cellular swelling, rapid release of tumor‐associated antigens, damage‐associated molecular patterns (DAMPs), and immune activating cytokine, which can not only modulate immunosuppression but also improve the immunogenicity.^[^ ^7^ ^]^ Studies have demonstrated the high efficiency of pyroptosis in antitumor immunity in vivo^[^ ^8^ ^]^ and the great potential of pyroptosis in anti‐recurrence.^[^ ^9^ ^]^ However, the exact role that pyroptosis plays in preventing postsurgical recurrence requires further investigation. Moreover, in the field of postoperative treatment, the modality of drug delivery plays a pivotal role, as it can significantly influence the efficacy and safety of the therapy. Studies have shown that injectable hydrogel is a kind of unique carrier with prominent biocompatibility, sustained drug release,^[^ ^10^ ^]^ and good postoperative site coverage, making it an ideal formulation for postoperative therapy.^[^ ^11^ ^]^

**Scheme 1:**
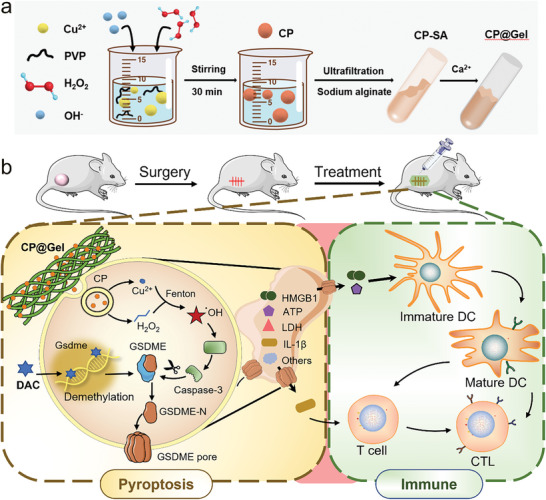
Schematic illustration of DAC+CP@Gel for inhibiting the postoperative recurrence of TNBC. a) Synthesis of CP@Gel. b) DAC+CP@Gel inhibits the postoperative recurrence of TNBC by inducing pyroptosis and anti‐tumor immune response.

Based on these facts, an injectable hydrogel‐encapsulated autocatalytic copper peroxide (CP@Gel) was fabricated in this study. The synthesized CP@Gel can generate adequate reactive oxygen species (ROS) spontaneously and independently of the H_2_O_2_ content at tumor sites, causing cellular oxidative stress and caspase‐3 activation for GSDME cleavage.^[^ ^12^ ^]^ However, *Gsdme* DNA is usually methylated in breast cancer cells,^[^ ^13^ ^]^ resulting in low GSDME levels. Hence, the DNA methyltransferase inhibitor decitabine (DAC) was utilized to inhibit *Gsdme* methylation and increase the expression of GSDME.^[^ ^14^ ^]^ Collectively, the DAC+CP@Gel achieved effective caspase‐3 activation and GSDME cleavage to induce intense pyroptosis and immune response that can inhibit postsurgical recurrence. The autocatalytic ROS nanoreactor can prevent drug resistance and unwanted side effects associated with chemotherapeutic drug‐induced pyroptosis. This innovative strategy kills residual tumor cells directly through pyroptosis while releasing the tumor‐associated antigens, adenosine triphosphate (ATP), high‐mobility group box protein 1 (HMGB1), immune activating cytokines interleukin‐1β (IL‐1β) and interferon‐γ (IFN‐γ), thus improving the immunogenicity and modulating immunosuppression to promote the maturation of DCs and the infiltration of cytotoxic T cells, resulting in the efficient repression of tumor growth and postoperative recurrence (**Scheme** **1**).

## 2 Results and Discussion

### 2.1 Preparation and Characterization of Copper Peroxide

**Figure 1:**
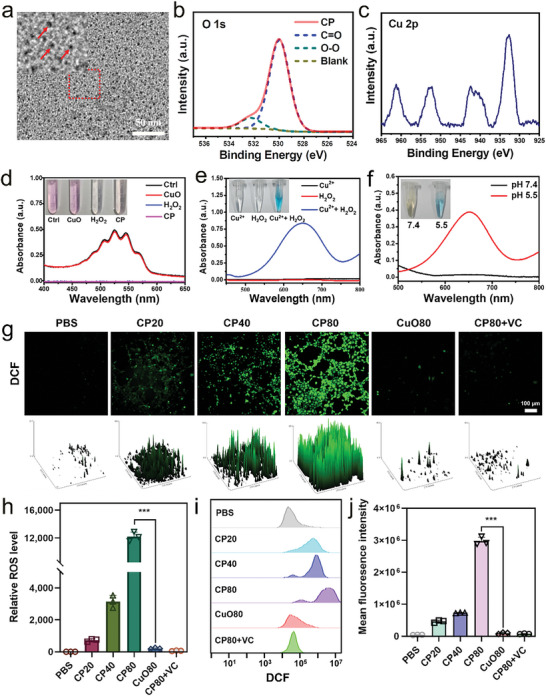
Synthesis and characterization of PVP‐coated CP. a) Representative TEM images of CP. b) High resolution O 1s XPS spectra obtained for CP. c) Cu 2p XPS spectra obtained for CP. d) Colorimetric analysis proving the presence of peroxo groups in CP. Peroxo groups reduce MnO4^−^ to colorless Mn^2+^ in strong acidic media (0.1 m H_2_SO_4_), so that the pink color disappears. e) UV absorption spectrum and photographs showing TMB aqueous solution treated with Cu^2+^, H_2_O_2_, or Cu^2+^+H_2_O_2_. f) Colorimetric detection of •OH produced by CP at different pH values based on TMB analysis. g) Fluorescence images and corresponding surface plots of 4T1 cells stained with DCFH‐DA probe after treatment with different concentrations of CP, CuO, or CP+VC over 4 h. h) Statistical results obtained for ROS levels based on fluorescence images. i) Flow cytometry results of 4T1 cells stained with DCFH‐DA following different treatments over 4 h. j) Histogram of mean fluorescence intensity from flow cytometry. CP20, CP40, CP80, and CuO80 in g–j represents 20 , 40 , and 80 µg mL^−1^ of CP or CuO, respectively. VC, vitamin C. Data are presented as the mean ± SD, n = 3, ^***^ *p* < 0.001.

To spontaneously generate an adequate amount of ROS, copper peroxide (CP) nanoparticles were first synthesized by reacting cupric chloride, poly(vinylpyrrolidone) (PVP), H_2_O_2_, and sodium hydroxide in an aqueous solution at room temperature for 30 min. Representative transmission electron microscopy (TEM) images indicated an average size of <10 nm for the prepared CP nanoparticles (**Figure** **1**a). X‐ray photoelectron spectroscopy (XPS) spectra showed two O 1s peaks at 532.5 and 530.0 eV, indicating O–O and C = O (Figure 1b), thus suggesting the presence of both peroxo groups and PVP. Two primary peaks in the Cu 2p XPS spectrum at 933.0 and 953.5 eV indicated a chemical valence of +2 for the Cu in the CP (Figure 1c). In addition, potassium permanganate‐mediated colorimetric analysis further confirmed the presence of peroxo groups in CP, which facilitated reduction of MnO_4_^−^ to colorless Mn^2+^ in the acidic media conditions, so that the color of permanganate disappeared when CP was added. In contrast, no color change was observed for copper oxide (CuO) (Figure 1d). To evaluate the ability of CP to produce •OH, the generation of •OH through a Fenton‐like reaction between Cu^2+^ and H_2_O_2_ was first determined by 3,3′,5,5′‐tetramethylbenzidine (TMB) analysis. This method was used because the highly active •OH can oxidize TMB to show a blue color with increased absorbance at 650 nm. The results showed that Cu^2+^ plus H_2_O_2_ did produce a rapid color change to blue in the TMB aqueous solution with a maximal absorption peak at 650 nm, while neither Cu^2+^ or H_2_O_2_ produced a blue color (Figure 1e). This indicated an excellent property of Cu^2+^ for Fenton‐like reaction to generate efficient •OH. The acid‐mediated decomposition of CP to Cu^2+^ and H_2_O_2_ resulted in an obvious color change in the TMB at pH 5.5, which was not observed under neutral conditions (pH 7.4) (Figure 1f), directly confirming the production of •OH by CP.

The •OH production ability of CP at the cellular level was further verified by dichlorofluorescein diacetate (DCFH‐DA) staining, which serves as an indicator for ROS. The results showed that 4T1 cancer cells that were incubated with CP displayed higher green fluorescence than those treated with PBS or CuO. Furthermore, the fluorescence signal increased alongside CP concentrations, and vanished when the antioxidant vitamin C (VC) was added (Figure 1g,h). Flow cytometry analysis produced results that were consistent with the fluorescence images (Figure 1i,j), indicating that the synthesized CP successfully generated abundant ROS.

### 2.2 Evaluation of Cytotoxicity and Pyroptosis of DAC+CP In Vitro

**Figure 2:**
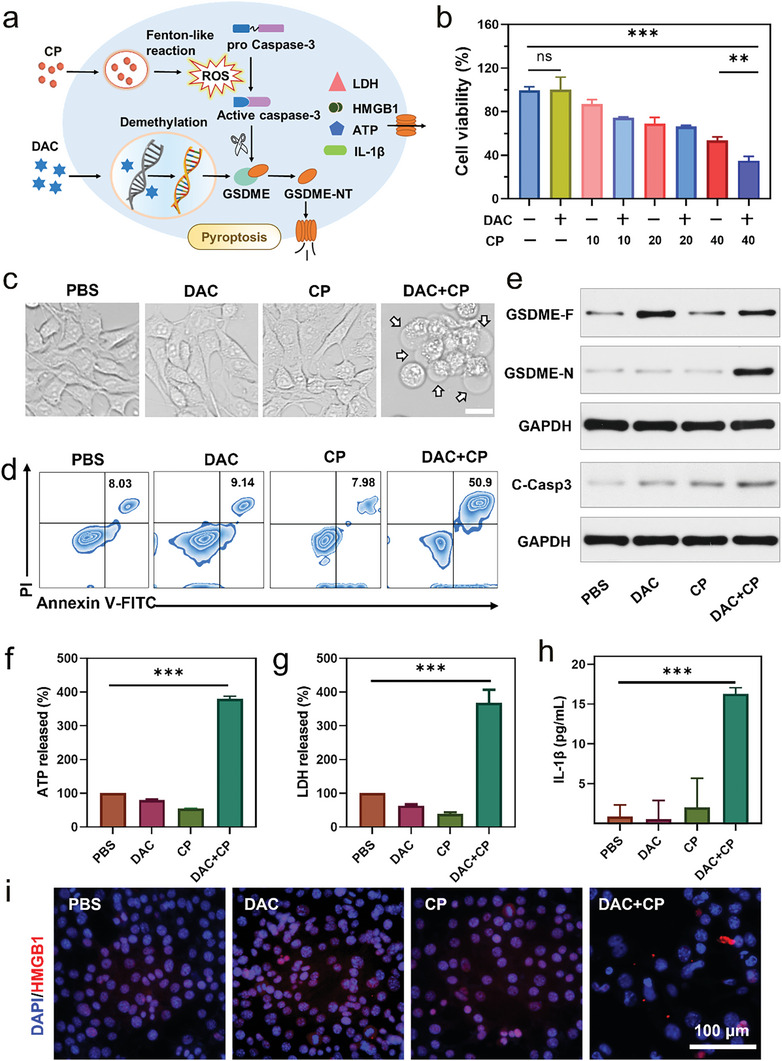
DAC+CP‐treated 4T1 cells undergoing pyroptosis. a) Schematic illustration of pyroptosis triggered by DAC and CP. b) Cell viability of 4T1 cells after treatment with 0.625 µm DAC and different concentrations of CP for 30 h, the unit is µg mL^−1^. c) Representative microscope images of 4T1 cells after treatment with PBS, DAC, CP, or DAC+CP for 6 h, 0.625 µm DAC, and 40 µg mL^−1^ CP were selected for here and subsequent experiments. d) Flow cytometric analysis for apoptosis and pyroptosis in 4T1 cells within 6 h of treatment based on Annexin V‐FITC and PI co‐staining protocol. e) Western blot analysis of GSDME‐F, GSDME‐N, and C‐Casp3 in 4T1 cells following different treatments. f–h) Release of ATP (f), LDH (g), and IL‐1β (h) in cells subjected to different treatments over 6 h. i) Cell immunostaining of HMGB1 in the nucleus after various treatments. Data are presented as the mean ± SD, n ≥ 3, ns = not significant, ^**^ *p* < 0.01, ^***^ *p* < 0.001.

As confirmed above, autocatalytic CP is able to produce enough ROS, which may activate caspase‐3 for GSDME cleavage. However, the pyroptosis executive protein GSDME is often absent or downregulated in patients with breast cancer (Figure S1a–c, Supporting Information) because of hypermethylation of the *Gsdme* gene. Therefore, DAC was used to pretreat tumor cells for demethylating *Gsdme* gene and upregulating the GSDME protein levels (**Figure** **2**a). To determine the optimal dose of DAC for breast cancer cell demethylation, 4T1 cells were treated with different concentrations of DAC for three days and the fold change of Gsdme mRNA was quantified by q‐PCR. The result showed an optimal concentration of 0.625 µm DAC for *Gsdme* gene demethylation in 4T1 cells (Figure S2a, Supporting Information). The GSDME protein levels were observed to consistently increase after DAC treatment (Figure S2b, Supporting Information). Thus, 0.625 µm of DAC was selected for subsequent experiments, and when combined with 40 µg mL^−1^ CP to treat 4T1 cells over 30 h, the cell viability decreased to ≈30% (Figure 2b), indicating efficient cell killing effect of DAC+CP.

Thereafter, the ability of DAC+CP to trigger pyroptosis was observed under an optical microscope, with results indicating that pyroptosis occurred rapidly, with initiation only 60 min after the addition of CP to the DAC‐pretreated cells (Figure S3a, Supporting Information). Typical morphological features of pyroptosis such as cell ballooning and swelling were observed in 4T1 cells treated with DAC+CP for 6 h, while minimal pyroptotic cell death was observed in cells treated with DAC or CP alone (Figure 2c; Figure S3b, Supporting Information). Next, the proportion of pyroptosis in differently treated cells were detected by flow cytometry after staining with Annexin V‐FITC and propidium iodide (PI). Compared with the PBS group, cells that were treated with CP and DAC+CP showed apparent programmed cell death, with the percentages of pyroptotic cell death significantly increased from 8% in the CP group to 51% in the DAC+CP group (Figure 2d; Figure S3c, Supporting Information). To further validate the pyroptosis process during treatment, western blot analysis was conducted. As shown in Figure 2e, the protein level of full‐length GSDME (GSDME‐F) was upregulated upon the treatment with DAC (DAC and DAC+CP group), and cleaved caspase‐3 (C‐Casp3) was increased in CP and DAC+CP group, while N‐terminal of GSDME (GSDME‐N) was significantly up‐regulated only in the DAC+CP group, indicating the co‐regulation of DAC and CP on GSDME cleavage and pyroptosis induction.

Subsequently, the release of intracellular content, including lactate dehydrogenase (LDH), ATP, IL‐1β, and HMGB1 was detected to further verify pyroptosis. The results in Figure 2f–g showed a considerably higher release of LDH and ATP in the supernatant of the DAC+CP‐treated cells, while no obvious release was observed in the other groups. Enzyme‐linked immunosorbent assay (ELISA) results demonstrated a dramatically increased IL‐1β concentration in the supernatant of the DAC+CP treated cells (Figure 2h). Consistently, immunostaining of HMGB1 in the nucleus after various treatments showed almost no HMGB1 signal in the nucleus after DAC+CP treatment, indicating the extracellular release of HMGB1 (Figure 2i; Figure S3d, Supporting Information). All above results were conducted in mouse cells, to lay the foundation for future clinical translation, DAC+CP‐treated experiments were further confirmed in human breast cancer cells MDA‐MB‐231. The typical pyroptotic morphology were observed in DAC+CP‐treated MDA‐MB‐231 cells (Figure S4a, Supporting Information). Cell viability measured using MTT assay also showed efficient cell killing effect of DAC+CP in human breast cancer cells (Figure S4b, Supporting Information). Additionally, the pyroptosis indicators such as LDH and ATP released in the supernatant of DAC+CP‐treated cells were much higher than those of other groups, indicating plasma membrane rupture and leakage after combined treatment (Figure S4c,d, Supporting Information). Collectively, these results indicate that DAC+CP could induce robust pyroptosis both in the mouse and human breast cancer cells in vitro.

### 2.3 Antitumor Effect of DAC+CP via Pyroptosis In Vivo

**Figure 3:**
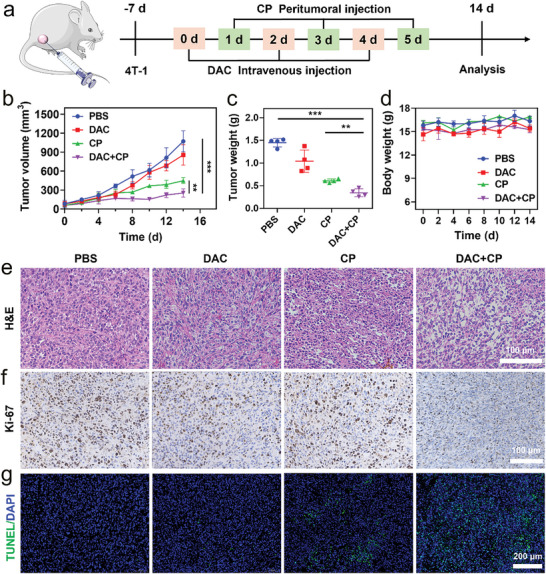
Antitumor effect of DAC+CP in vivo. a) Schematic illustration of 4T1 breast cancer model and treatment process. BALB/c female mice with an intact and functional immune system were subcutaneously inoculated with 4T1 cells. b) Tumor volume in various groups. c) Quantitative analysis of tumor weight 14 d after different treatments. d) Body weights in different groups. (e–g) H&E, Ki67, and TUNEL staining of tumor tissues after different treatments. Data are presented as the mean ± SD, n = 4, ^**^ *p* < 0.01, ^***^ *p* < 0.001.

Considering the favorable antitumor activity of DAC+CP in vitro, the antitumor effect of DAC+CP was then evaluated in vivo. The immunocompetent BALB/c female mice were subcutaneously inoculated with 5 × 10^5^ 4T1 cells at the right flank to build a subcutaneous tumor model. One week after 4T1 tumor cell inoculation, mice were randomly divided into four groups and treated with PBS, DAC, CP, or DAC+CP (n = 4). DAC was administered intravenously on days 0, 2, and 4, and CP was administered via peritumoral injection on days 1, 3, and 5 (**Figure** **3**a). The tumor volume and body weight of the mice were measured every two days. The results showed that treatment with DAC or CP alone resulted in limited antitumor effects, while the DAC+CP treatment showed significant antitumor effects (Figure 3b,c) with no obvious changes in body weight (Figure 3d). The corresponding tumor photographs further showed that DAC+CP was the most effective treatment (Figure S5, Supporting Information). Hematoxylin and eosin (H&E) and Ki67 staining of tumor tissue confirmed the bigger tumor destruction and proliferation inhibition for the DAC+CP group, and terminal‐deoxynucleoitidyl transferase mediated dUTP nick end labeling (TUNEL) indicated more apoptotic cells in the DAC+CP treatment group (Figure 3e–g). Finally, histological staining indicated no detectable damage in the hearts, livers, spleens, lungs, or kidneys of the mice after the different treatments (Figure S6, Supporting Information), indicating that DAC+CP is a safe and effective platform for TNBC treatment.

### 2.4 DAC+CP Stimulates the Antitumor Immune Response

**Figure 4:**
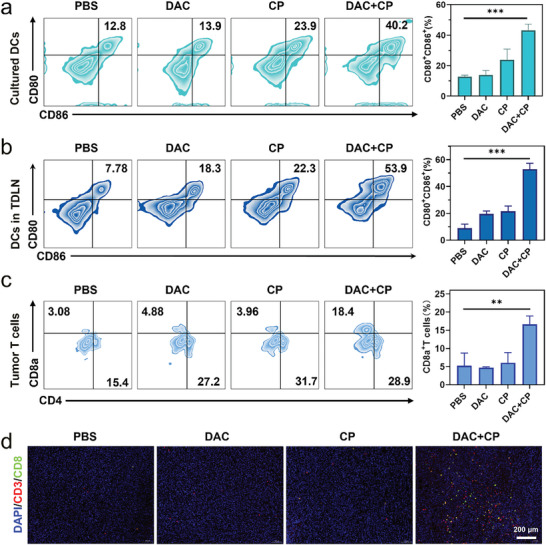
Antitumor effect of DAC+CP via the pyroptosis‐evoked immune response. a) Flow cytometric analysis and quantification of CD80^+^CD86^+^ DCs after co‐culture with supernatant collecting from different groups. b) Flow cytometric analysis and the statistical result of CD80^+^CD86^+^ dendritic cells in tumor‐draining lymph node (TDLN). c) Flow cytometric analysis and quantification of CD8a^+^ T cells in tumors. d) Immunofluorescence staining of CD3 and CD8 of tumor tissues after different treatments. Data are presented as the mean ± SD, n≥3, ^**^ *p* < 0.01, ^***^ *p* < 0.001.

To investigate whether the immunogenic pyroptosis can evoke antitumor immune responses in vivo, we first examined whether the treatment can induce DCs maturation in vitro by co‐culturing bone marrow‐derived DCs with the supernatant collecting from 4T1 cells with different treatments. The results showed high expression of CD80 and CD86 on the surface of the DCs after coculture with the supernatant from DAC+CP group, indicating DAC+CP treatment promoted DCs activation and maturation (**Figure** **4**a). Since DCs process and present antigens to CD4^+^ T cells in the lymph nodes, we next examined the activation of DCs in tumor‐draining lymph node (TDLN). Flow cytometry results showed that the DAC+CP treatment could dramatically induce DCs maturation (CD80^+^CD86^+^), the percentage significantly increased compared with the PBS group (Figure 4b). Further quantitative flow cytometry of tumor T cells showed a remarkably increase in the presence of CD8a^+^ T cells in the tumor microenvironment of the DAC+CP group (Figure 4c). Finally, immunofluorescence staining showed significant enhancement of the CD3^+^ and CD8^+^ T cells under DAC+CP administration (Figure 4d). All these results proved that cancer cell pyroptosis, that is induced by DAC+CP, can induce DCs maturation and T cell activation for solid tumor immunotherapy.

### 2.5 DAC+CP@Gel Prevents Postoperative Recurrence of TNBC

**Figure 5:**
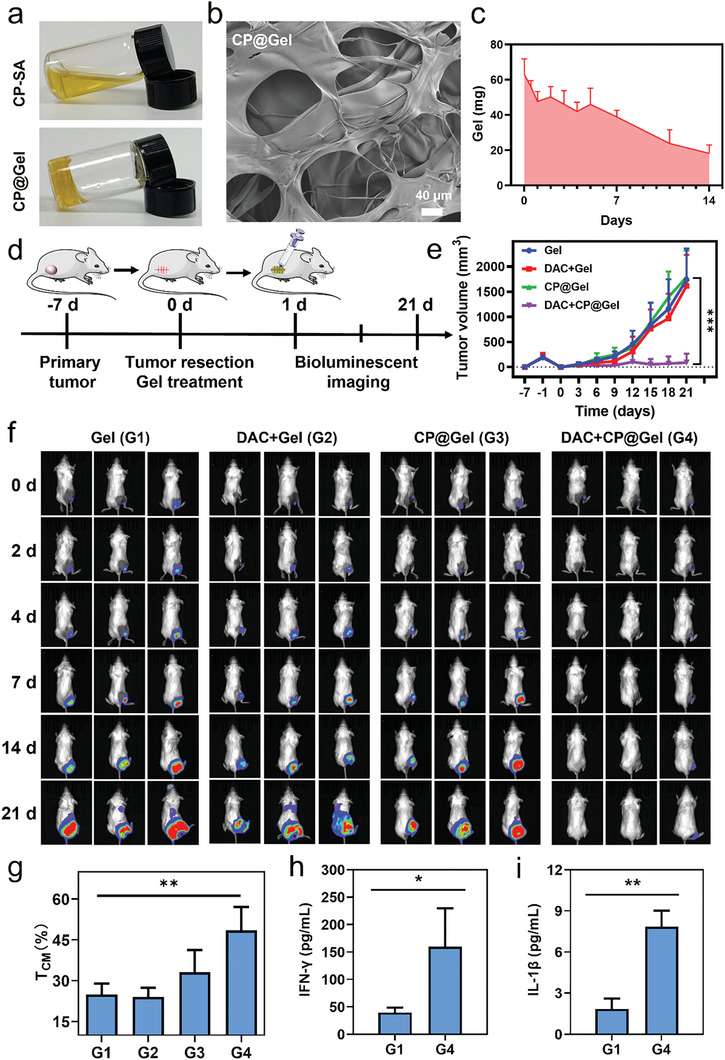
DAC+CP@Gel effectively suppresses tumor recurrence in 4T1 tumor resection model. a) Digital photographs of temperature induced reversible sol‐gel transition of CP‐SA and CP@Gel. b) TEM image showing backbone structure of the CP@Gel. c) The remaining mass of CP@Gel in vivo. d) Schematic representation showing 4T1 tumor resection model and treatment process. e) Tumor volume in different groups. f) In vivo bioluminescence images of mice after different treatments. g) Statistic result of T_CM_ in spleen by flow cytometry. h,i) IFN‐γ and IL‐1β cytokines in serum. Data are presented as the mean ± SD, n ≥ 3, ^*^ *p* < 0.05, ^**^ *p* < 0.01, ^***^ *p* < 0.001.

To achieve sustained drug release for inhibiting the postoperative recurrence of TNBC, CP@Gel was fabricated. Briefly, CP nanoparticles were dissolved in sodium alginate solution (SA), resulting in a clear yellow solution that converted instantly to hydrogel following the addition of Ca^2+^, due to the chelation of Ca^2+^ and SA (**Figure** **5**a). The effects of CP particles on sodium alginate and gelation were explored, the results showed that copper peroxide particles do not decompose and release Cu^2+^ under our experimental conditions in the extracellular environment, do not react with sodium alginate and have no effect on the gelation (Figure S7a,c, Supporting Information). Next, the morphology of the CP@Gel was characterized using TEM. Figure 5b shows that the hydrogel was in the form of plate‐like particles, which are the typical backbone structures of SA‐based hydrogels. Because of Ca^2+^ abundance at the tumor site, the CP@Gel system could be used as an injectable hydrogel for intra‐tumoral administration to suppress tumor recurrence. Studies have shown that the in vivo biodegradation of SA‐based hydrogels is slow and can be maintained in postoperative local tissues for long periods due to its good biocompatibility. Therefore, the biodegradation of the CP@Gel injectable hydrogel was evaluated after in vivo gelatinization. An average mass of ≈60 mg CP@Gel was obtained at the time of gel formation, and the hydrogel continued to decompose in vivo, with the average mass of the hydrogel decreasing to 38.9 mg, ≈64.8% of the original, after seven days, and to 18.3 mg, ≈30.5% of the original, after 14 days (Figure 5c), indicating that the injectable CP@Gel system can degrade slowly for more than two weeks.

Next, a subcutaneous breast tumor recurrence model was established to further validate the potential anti‐recurrence efficacy of DAC+CP@Gel in vivo. BALB/c female mice were subcutaneously inoculated with 5 × 10^5^ 4T1‐Luc cells in 100 µL PBS per mouse. When reached an average size of 100 mm^3^, the tumors were removed while a layer of surrounding skin was left to allow for tumor recurrence (Figure 5d). After surgery, mice were treated once with the different formulations and tumor recurrence and growth were monitored. The results showed rapid local regrowth in all mice in the Gel, DAC+Gel, and CP@Gel groups, while local tumor recurrence and regrowth was significantly decreased in the DAC+CP@Gel treatment group, only one out of three mice had a minor recurrence, with 67% elimination (Figure 5e,f). These results demonstrate the greatest anti‐recurrence effect for DAC+CP@Gel in the TNBC recurrence model. To explore whether DAC+CP@Gel therapy could generate memory T cells, CD44 and CD62L expression on T cells was used to detect central memory T cells (T_CM_), with results showing an increase in the CD62L^+^CD44^+^ T_CM_ following DAC+CP@Gel therapy (Figure 5g). Additionally, the results of ELISA for quantitative detection of the pro‐inflammatory cytokine IL‐1β and T cell function marker IFN‐γ showed significantly elevated concentrations after treatment with DAC+CP@Gel (Figure 5h,i). These observations collectively demonstrate that the DAC+CP@Gel prominently enhances the antitumor immune response, indicating its use as a safe and promising platform for inhibiting the postsurgical recurrence of TNBC.

## 3 Conclusion

Here, we report an innovative therapeutic platform that integrates sustained drug release, autocatalysis, and epigenetic modification to effectively inhibit TNBC growth and recurrence. Specifically, injectable hydrogel‐encapsulated autocatalytic CP nanoparticles (CP@Gel) and DNA methyltransferase inhibitor DAC were used for pyroptosis induction and immune activation. This strategy not only kills residual cancer cells, bypassing apoptosis resistance, but also modulates the ITME to activate the anti‐tumor immune response. After local injection, the CP@Gel released CP and autocatalytically generated ROS for caspase‐3 activation, and the systematically administered DAC was able to inhibit the methylation of *Gsdme* to elevate the GSDME protein level, eventually resulting in robust pyroptosis and an anti‐tumor immune response that inhibited TNBC proliferation and postoperative recurrence. The results demonstrated a significant decrease in the local tumor recurrence owing to the DAC+CP@Gel treatment, with 67% elimination. Although there are potential challenges may face in clinical settings, our study presents promising preclinical findings and lays a foundation for future translation. In future, the drug safety and effectiveness must be further evaluated in clinical trials, it is essential to establish the optimal dosage and administration regimen to minimize potential side effects while maintaining therapeutic efficacy, and patient variability should be taken into consideration. In summary, this work suggests considerable potential for the pyroptosis‐based and injectable hydrogel‐aided strategy in preventing the postoperative recurrence of TNBC.

## 4 Experimental Section

### Synthesis of CP

The CP was synthesized as described previously^[^ ^12a^ ^]^ with modifications. Briefly, 681.92 mg CuCl_2_·2H_2_O (4 mm), 160 mg NaOH (4 mm) was dissolved in 4 mL water, respectively, to prepare two mother fluids with a concentration of 1m. PVP (0.5 g) was dissolved in 8 mL H_2_O and then added to prepared CuCl_2_ (50 µL, 1 m). NaOH (100 µL, 1 m) and H_2_O_2_ (100 µL, 35%) were diluted to 2 mL and then added dropwise to the above mixture under stirring. After further stirring for 30 min, the PVP‐coated CP nanoparticles were collected by ultrafiltration and washed with water several times, and then dissolved in 4 mL water for use.

### Preparation of the CP@Gel

The prepared CP nanoparticles were dissolved in sodium alginate (SA) solution to obtain yellow solution. Then, use a syringe to absorb the CP‐SA and slowly inject into CaCl_2_ solution drop by drop. Due to the chelation of Ca^2+^ and carboxyl group of SA, the yellow solution was transformed into the gel state (CP@Gel) instantly.

### Colorimetric Assay of Peroxo Groups

KMnO_4_ (50 µg mL^−1^) was dissolved in 0.1 m H_2_SO_4_. The mixture was treated with CP, CuO, H_2_O_2,_ or H_2_O for 10 min at room temperature. And then, the UV–vis spectra were measured from 400 to 650 nm.

### Production of •OH by Cu^2+^‐Driven Fenton‐Type Reaction

The acetate buffer solution (pH 5.5) containing TMB (100 µg mL^−1^) were mixed with Cu^2+^ (100 mm), H_2_O_2_ (100 mm) or Cu^2+^+H_2_O_2_, respectively. The generation of •OH was determined by the absorption increase at 650 nm. The Cu^2+^ or H_2_O_2_ alone were used as control groups.

### pH‐Dependent •OH Generation by CP Nanoparticles

The CP nanoparticles (0.2 mg mL^−1^) were added to the TMB solutions with different pH values (7.4 or 5.5). After mixing for 2 h, the absorption spectra were measured.

### Cell Culture

Mouse breast cancer cell 4T1 was cultured in RPMI 1640 medium, human breast cancer cell MDA‐MB‐231 was cultured in DMEM medium containing 1% antibiotics (penicillin‐streptomycin, 10 000 U mL^−1^) and 10% heat‐inactivated FBS. Both cells were cultured with an atmosphere of 5% CO_2_ at 37 °C.

### Intracellular ROS Generation Assay

DCFH‐DA was employed as a fluorescent ROS probe to indicate CP nanoparticles‐induced oxidative stress. Briefly, 4T1 cells were exposed to CP nanoparticles with different concentrations (0, 20, 40, and 80 µg mL^−1^) for 4 h. Then, the cells were stained with DCFH‐DA (10 µm) for 30 min, the fluorescence images were recorded and flow cytometric analysis was conducted.

### Quantitative Real‐Time PCR

The level of demethylated *Gsdme* in tumor cells of differently treated groups were determined by q‐PCR. RNA was isolated from cells with Trizol reagent and dissolved with RNase‐free water. The first‐strand synthesis of cDNA was performed by Prime Script RT reagent Kit with gDNA Eraser. Then, 1 µL of RNA sample was used in a subsequent fluorescence quantitative PCR experiment run according to the manufacturer's parameters (SYBR Premix Ex Taq) on a Step One Real‐Time PCR machine (Life technologies). The reaction procedure was as follows: 95 °C for 1 min; 95 °C for 15 s, 58 °C for 20 s, 72 °C for 45 s, 40 cycles; 60  to 95 °C, raised the temperature by 1 °C every 20 s. The demethylated *Gsdme* expression was calculated by ΔΔCT method. The used primers are as follows:

Gsdme (TGATTAGCGGCAGGCCTAAG, GCTCCAGATTATCCGGGAGATG);

Gapdh (CAGGAGGCATTGCTGATGAT, GAAGGCTGGGGCTCATTT).

### Western Blot Assay

GSDME‐F, GSDME‐N, and C‐Casp3 expression in 4T1 cells after different treatments were tested by Western blot. All the treated cells were washed and lysed to extract protein. The samples were heated for 5 min and separated on an SDS‐PAGE. After electrophoresis, the isolated proteins were transferred to the PVDF membrane. The membrane was blocked with 5% skimmed milk for 1 h and incubated primary antibody overnight at 4 °C. Subsequently, the membrane was incubated with the corresponding secondary antibody labeled with horseradish peroxidase for 1 h at room temperature. At last, the protein bands under different conditions were visualized using ultrasensitive enhanced chemiluminescence reagent and analyzed using Fiji.

### Annexin V‐FITC/PI Assay

The cell apoptosis and pyroptosis assay of 4T1 cells was performed to evaluate the death mechanism of different samples. 4T1 cells were seeded into 6‐well plates and incubated for 24 h. Then the cells were co‐incubated with PBS, DAC (0.625 µm, 3 days), CP (40 µg mL^−1^, 6 h) or DAC+CP in culture medium. After incubated in fresh medium for another 24 h, the cells were stained with Annexin V‐FITC/PI according to the instruction of the kit and then analyzed through fluorescence activating cell sorter (FACS). Fluorescence emission of Annexin V‐FITC and PI were respectively received by FL1 (Ex: 488 nm, Em: 500–540 nm) and FL2 (Ex: 488 nm, Em: 570–680 nm) channel.

### LDH and ATP Release Assay

The amounts of LDH in cell supernatant was determined by colorimetry based on an INT chromogenic reaction catalyzed by diaphorase with LDH Release Assay Kit (Beyondtime). 4T1 cells were seeded into a 96‐wells plate and incubated for 24 h. Then the cells were co‐incubated with PBS, DAC (0.625 µm, 3 days), CP (40 µg mL^−1^, 6 h) or DAC+CP in fresh media. LDH release reagents was added to the maximum enzyme activity sample control wells (untreated cells for subsequent lysis) by 10% of the volume of original medium 1 h before the measurement. Then, the cells were centrifuged at 400 g for 5 min to obtain the supernatant. Subsequently, 120 µL of the supernatants were added into corresponding wells of a new 96‐wells plate. Then 60 µL of LDH detection reagents was added to each well and incubated in the dark for 30 min after blending. The absorbance was measured at 490 nm. The LDH release were calculated as follows: (drug‐treated sample absorbance – control sample absorbance)/(the maximum enzyme activity sample control absorbance – control sample absorbance). For ATP detection, 100 µL of ATP detection reagents (Beyondtime) was added to wells and incubated 5 min at room temperature. Then 20 µL of samples or standard solutions were added to each well and mixed quickly. The luminescence of the samples was measured by a Lumat LB 9507 (Berthold, Germany) for ATP calculation.

### Enzyme‐Linked Immunosorbent Assay (ELISA) Analysis

The pro‐inflammatory molecules IL‐1β in cell culture medium suspension of differently treated groups were detected by ELISA. The differently treated cells were centrifuged at 100 g for 5 min to obtain the supernatants. Then 100 µL samples or prepared standard solution at different concentrations were added to corresponding wells of the 96‐well plate (specific monoclonal antibody of target protein had been coated) and incubated for 2 h at room temperature. After washing, 100 µL of biotinylated antibody was added to each well and incubated for 1 h at room temperature. Subsequently, 100 µL of horseradish peroxidase‐labeled streptavidin and TMB substrate were added to each well in turn and incubated for 20 min at room temperature in the dark, respectively. Finally, 50 µL of stop solution was added to each well and the optical density (OD) value at 450 nm was measured immediately after mixing.

### In Vitro DCs Maturation Assay

Bone‐marrow‐derived dendritic cells (BMDCs) were prepared and seeded in 6‐well plate. After further incubated with different formulations‐treated 4T1 cell supernatant for 24 h, BMDCs cells were collected and incubated with fluorescent anti‐CD11c, anti‐CD80, and anti‐CD86 antibodies for flow cytometric analysis.

### Flow Cytometry

The collected tumor draining lymph node (TDLN), tumors, and spleen tissues were used for the analysis of immunocytes via FACS. Briefly, TDLN were collected and prepared in single‐cell suspensions for detection of mature DCs, CD80^+^CD86^+^ cells gating on CD11c^+^ cells were analyzed and quantified. The tumor tissues were digested and analyzed the CD8a^+^ T cells, the initial step involves applying a specific gating strategy to exclude adherent materials and multimers; subsequently, the CD3‐positive population is gated, and within this group, the CD4‐positive or CD8a‐positive cell subsets are identified. Fresh spleen tissues were collected for memory T cell analysis. The spleen was digested by 1 mg mL^−1^ collagenase IV, containing 0.1 mg mL^−1^ hyaluronidase and 0.1 mg mL^−1^ DNase I for 1 h at 37 °C. The suspension was then filtered through a 70 µm nylon cell‐strainer and centrifuged. CD8, CD62L, and CD44 antibodies were incubated with the spleen cells for 20 min. The cells were washed with PBS to remove excess antibody for followed detection by flow cytometry.

### Immunofluorescence Staining

After treatment, all mice were sacrificed and tumors were collected and fixed in 4% paraformaldehyde, and sectioned after paraffin embedding. The tumors tissue was stained with H&E, Ki67, TUNEL, and immunofluorescence staining for CD3^+^ and CD8^+^ T cells. Major organs including heart, liver, spleen, lung, and kidney tissues of mice in each group were stained with H&E and observed by microscope.

### In Vivo Antitumor Efficacy

BALB/c female mice (4–5 weeks) were purchased from Beijing HFK Bio‐Technology co. LTD. All procedures were approved by the Institutional Animal Care and Use Committee of the Fourth Military Medical University (Xi'an, China. Approval/accreditation number: 20 230 466). The tumor recurrence model was established by subcutaneous injection of 5×10^5^ 4T1‐Luc cells in 100 µL PBS per mouse. When the tumor reached an average size of 100 mm^3^, ≈95% of total tumor was resected and the bioluminescence signal of remaining tumor tissue was detected by IVIS. Subsequently, mice were randomly divided into four groups and resection cavities were filled with various formulations, including Gel, DAC+Gel, CP@Gel, and DAC+CP@Gel. The Gel, DAC or CP@Gel were administrated every day for total 3 days, respectively. Boy weight and tumor volume were monitored every two days throughout the experiment.

### Statistical Analysis

The data are presented as the mean±SD based on at least three independent experiments (n ≥ 3). Statistical significance was determined using two‐tailed unpaired Student's t‐test or by ANOVA for comparison between multiple groups. *p* < 0.05 were considered as statistically significant (^*^ *p* < 0.05, ^**^ *p* < 0.01, and ^***^ *p* < 0.001). The statistical significance was analyzed using GraphPad Prism 9.5.1 Software.

## Conflict of Interest

The authors declare no conflict of interest.

## Supporting information



Supporting Information

## Data Availability

The data that support the findings of this study are available from the corresponding author upon reasonable request.
