# Supplementary material for: Injectable Autocatalytic Hydrogel Triggers Pyroptosis to Stimulate Anticancer Immune Response for Preventing Postoperative Tumor Recurrence
Source: Adv Sci (Weinh). 2024 Oct 28;12(1):2408415. doi: 10.1002/advs.202408415 (PMC11714207; doi:10.1002/advs.202408415)
Supplement: Supplementary file 1 — Supporting Information [file ADVS-12-2408415-s001.docx]

Supporting information

**Injectable Autocatalytic Hydrogel Triggers Pyroptosis to Stimulate Anticancer Immune Response for Preventing Postoperative tumor Recurrence**

*Zhiping Rao^†^, Yutong Zhu^†^, Zhuang Chen^†^, Yi Luo, Zuo Yang, Weijing Liu, Chaoqiang Qiao, Yuqiong Xia, Peng Yang^*^, Dong-Man Ye^*^, Zhongliang Wang^*^*


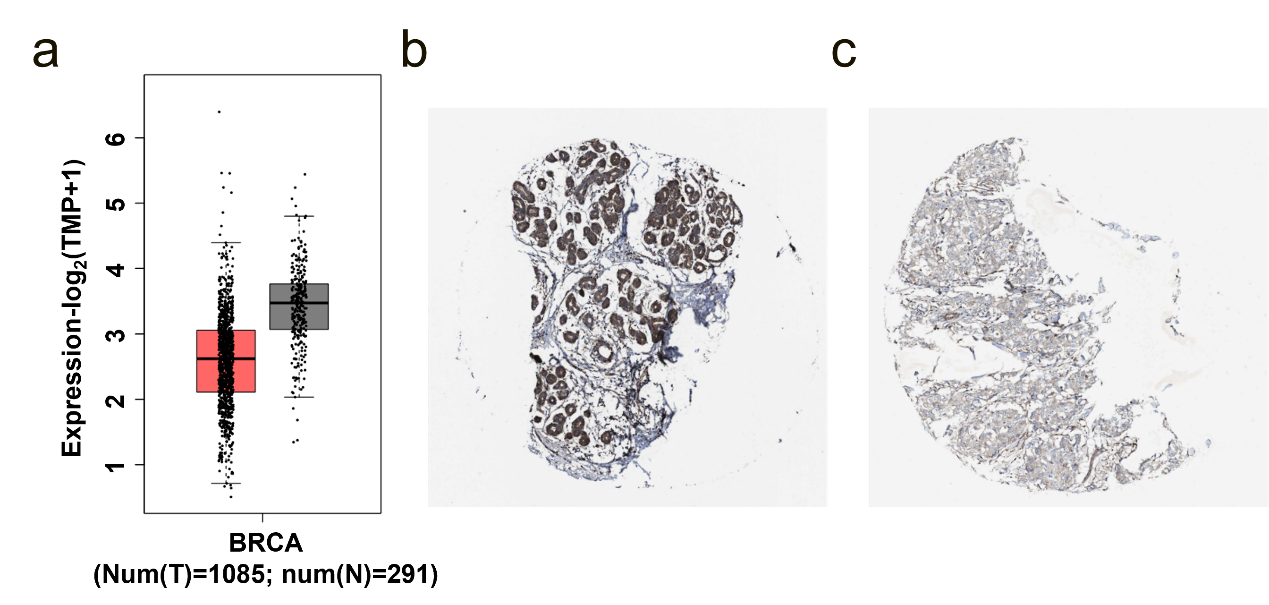


**Figure S1**. The analysis of GSDME expression in breast cancer cell (BRCA) patients. (a) The TCGA database of GSDME expression in BRCA patients. The boxplot analysis showing log_2_ (TPM+1) on a log-scale. (b-c) Immunohistochemistry of clinical samples of breast cancer tissue or normal breast tissue.


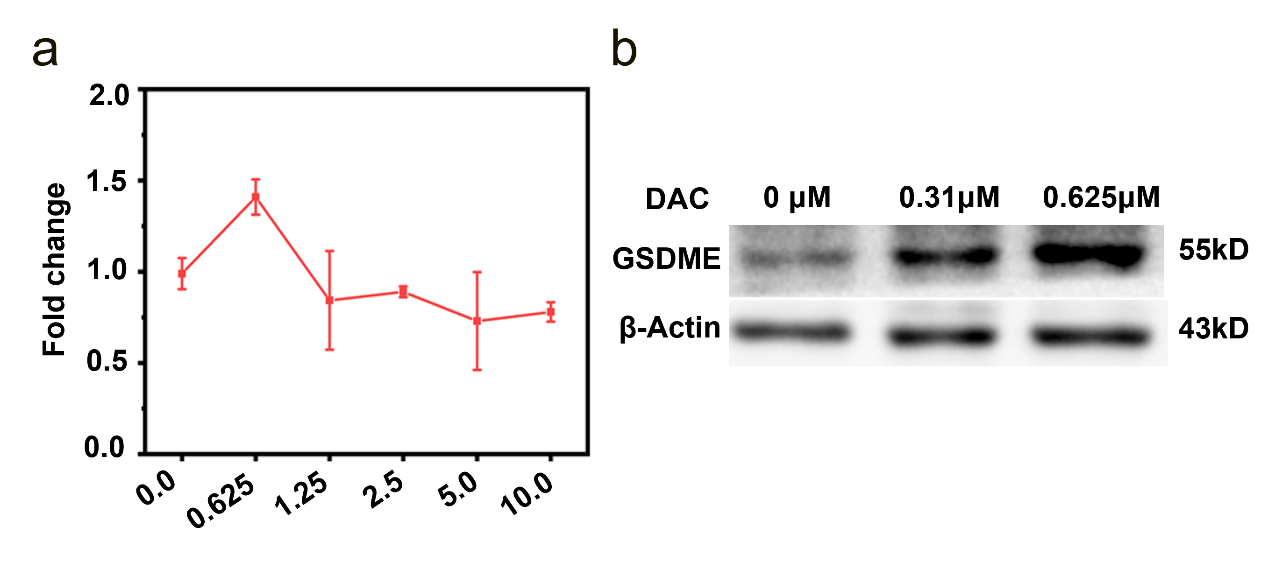


**Figure S2**. DAC treatment increase the GSDME expression in 4T1 cells. (a) q-PCR analysis of relative *Gsdme* mRNA level in 4T1 cells after treated by diﬀerent concentrations of DAC. The unit is μM in X axis. (b) Western blotting analysis of GSDME expression in 4T1 cells treated by DAC.


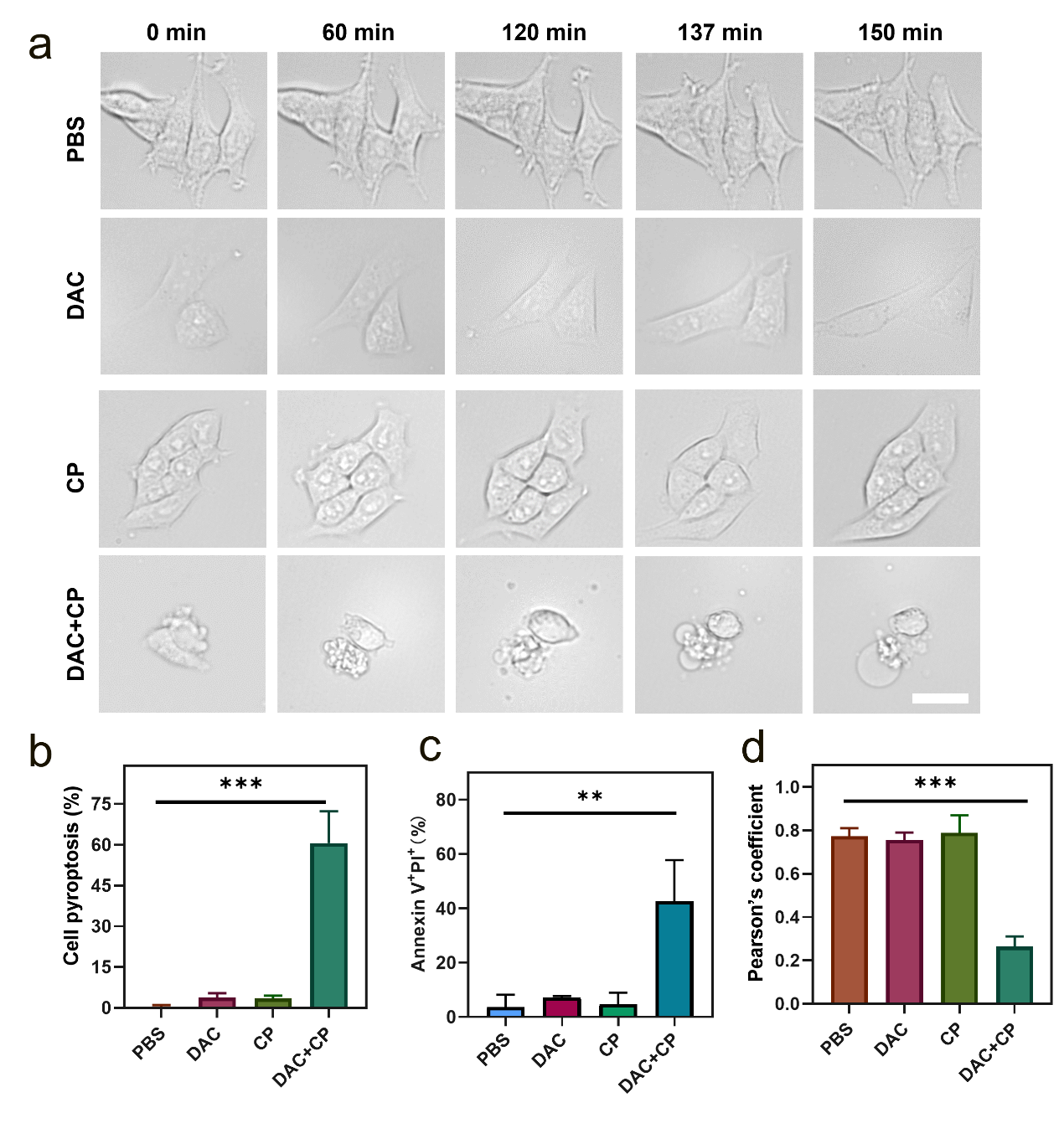


**Figure S3**. (a) Time lapse microphotographs of 4T1 cells after different treatments, which shows the process of pyroptosis. Scale bar: 25 μm. (b) The proportion of pytoptosis cells under microscopic observation after different treatments. (c) Statistical results for apoptosis and pyroptosis in 4T1 cells within 6 h of treatment based on Annexin V-FITC and PI staining and flow cytometry. (d) Fluorescence co-localization analysis of HMGB1 staining and DAPI nuclear staining after various treatments. The Pearson’s coefficient is used to measure the degree of co-localization; a value closer to 1 indicates a stronger positive correlation, suggesting a high degree of co-localization between HMGB1 and DAPI. Conversely, a value closer to 0 indicates a lack of correlation, implying low co-localization and suggesting that HMGB1 has translocated out of the nucleus.


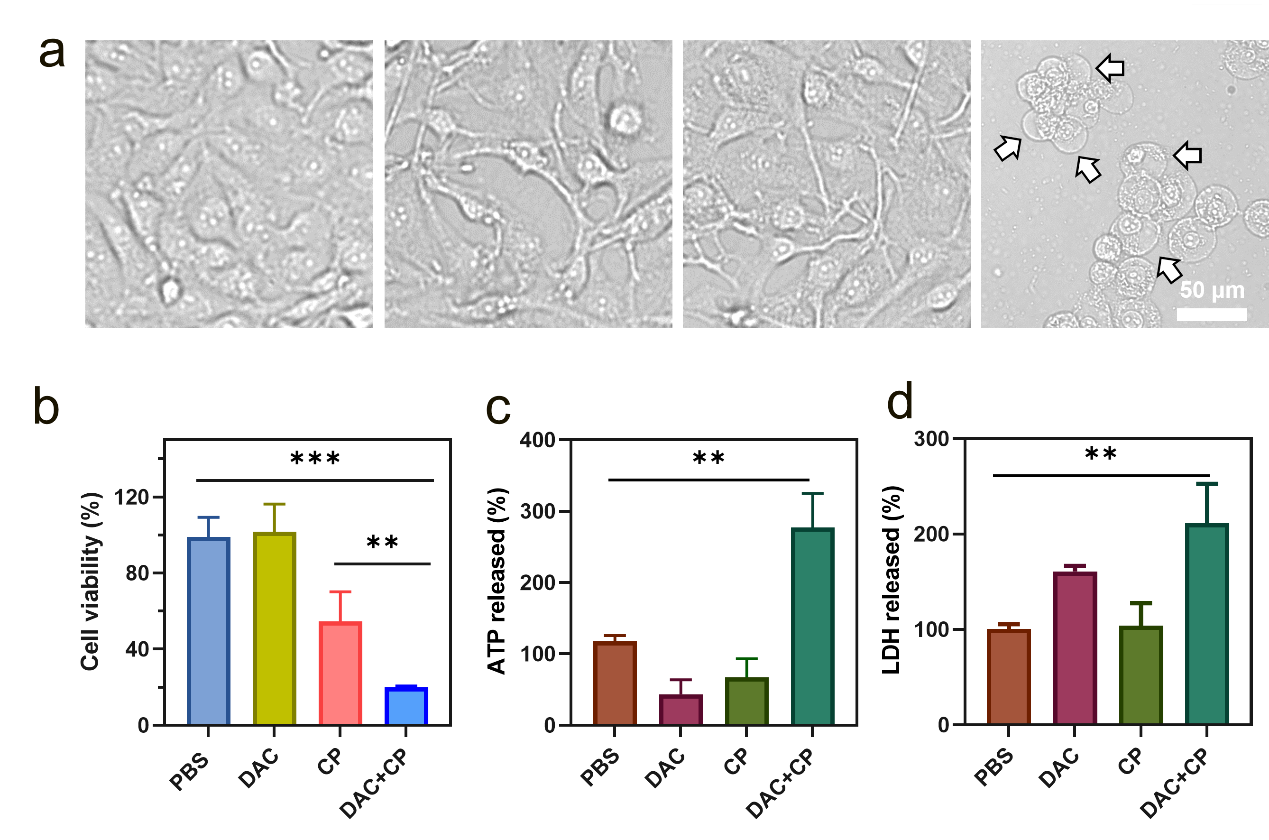


**Figure S4.** DAC+CP treated MDA-MB-231 cells undergoing pyroptosis. (a) Representative microscope images of MDA-MB-231 cells after treatment with PBS, DAC (0.625 μM), CP (40 μg mL^−1^), or DAC+CP for 6 h. (b) Cell viability of MDA-MB-231 cells after different treatment for 30 h. (c-d) Release of ATP (c) and LDH (d) in cells subjected to diﬀerent treatments over 6 h. Data are presented as the mean ± SD, n≥3, ** *p* < 0.01, *** *p* < 0.001.


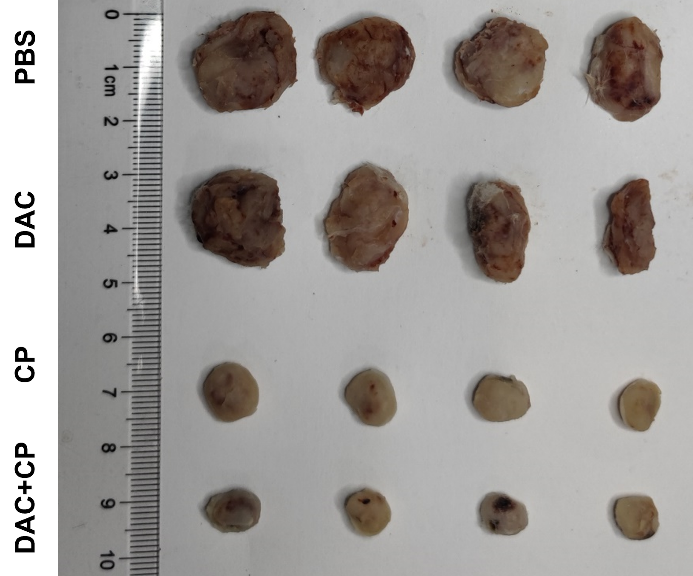


**Figure S5**. The photographs of the tumors post different treatments.


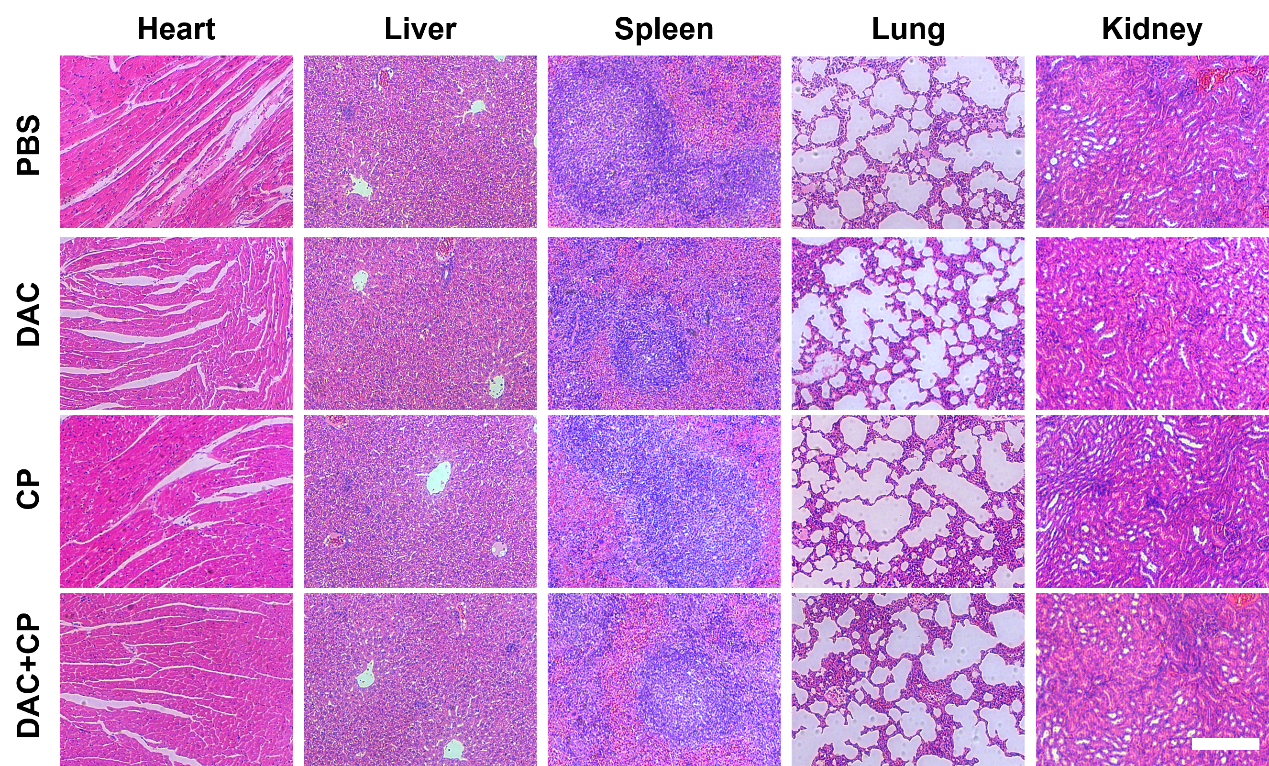


**Figure S6**. H&E staining indicated minimal lesions to heart, liver, spleen, lung and kidney both in different groups. Scale bar: 200 μm.


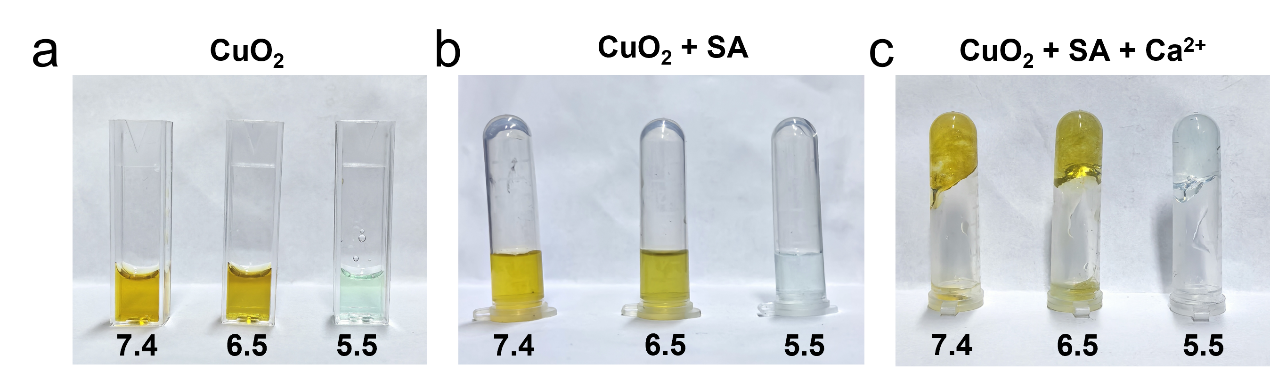


**Figure S7.** Effects of copper peroxide particles on sodium alginate and calcium alginate hydrogels. (a) The state of copper peroxide particles in solutions with different pH values. (b) The effect of copper peroxide particles on sodium alginate solutions under different pH conditions. (c) The state of copper peroxide particles and its effect on calcium alginate gels under different pH conditions.
